# B-mode ultrasound characteristics of carotid plaques in symptomatic and asymptomatic patients with low-grade stenosis

**DOI:** 10.1371/journal.pone.0291450

**Published:** 2023-09-13

**Authors:** Salahaden R. Sultan, Mohammed Khayat, Bander Almutairi, Abdulhamid Marzouq, Ahmad Albngali, Rawan Abdeen, Adnan A.S. Alahmadi, Fadi Toonsi

**Affiliations:** 1 Radiologic Sciences, Faculty of Applied Medical Sciences, King Abdulaziz University, Jeddah, Saudi Arabia; 2 Department of Radiology, King Abdulaziz University Hospital, Jeddah, Saudi Arabia; 3 Department of Radiology, Faculty of Medicine, King Abdulaziz University, Jeddah, Saudi Arabia; Assiut University, EGYPT

## Abstract

Carotid plaque features assessed using B-mode ultrasound can be useful for the prediction of cerebrovascular symptoms. Therefore, the aim of this retrospective study was to determine the ability of ultrasound B-mode imaging to differentiate between carotid plaques causing less than 50% stenosis in symptomatic and asymptomatic patients. A dataset of 1,593 patients with carotid disease who underwent carotid ultrasound between 2016 and 2021 was evaluated retrospectively between January and April of 2022. A total of 107 carotid plaques from 35 symptomatic and 52 asymptomatic patients causing low-grade stenosis on B-mode images were included in the analysis. Chi-square, independent t-test and Mann-Whitney U test were used to compare the variables. There was a significant association between hypertension and the presence of cerebrovascular symptoms (p = 0.01). Predominantly hypoechoic and hyperechoic carotid plaque were significantly associated with the presence and absence of cerebrovascular symptoms, respectively (predominantly hypoechoic: p = 0.01; predominantly hyperechoic: p = 0.02). Surface irregularity was significantly associated with the presence of cerebrovascular symptoms (p = 0.02). There is was a significant difference in the carotid plaque length and area between the symptomatic and asymptomatic patients (plaque length: symptomatic median 9 mm, interquartile range [IQR] 6 mm; asymptomatic median 6 mm, IQR 4.5 mm, p = 0.01; plaque area: symptomatic median 24 mm, IQR 30 mm; asymptomatic median 14 mm, IQR 17 mm, p = 0.01); however, this difference was not significant for plaque thickness (p = 0.55), or common carotid artery intima-media thickness (p = 0.7). Our findings indicate that hypertension patients with predominantly hypoechoic carotid plaques and plaques with an irregular surface are associated with the presence of cerebrovascular symptoms. In addition, the carotid plaques in symptomatic patients were longer and larger compared to asymptomatic patients.

## Introduction

Stroke is a major health concern, and strokes can be classified as hemorrhagic type, which is attributed to blood vessel rupture and represents 38% of strokes, or ischemic type, which is caused by interruption of the blood supply to the brain and accounts for 62% of strokes [1, 2]. Risk factors associated with stroke include age, gender, hypertension, diabetes mellitus and smoking cigarette [3]. According to Global Burden of Diseases Study, there were more than 12 million cases of stroke in 2019, causing 143 million disabilities and 6.55 million deaths [1]. The same study reported that from 1990 to 2019 the incidence of stroke increased by 70%, and disabilities and deaths from stroke increased by 32% and 43%, respectively. Therefore, investigating effective and reliable biomarkers for stroke is important.

Atherosclerosis is a chronic inflammatory disease characterized by lipid accumulation, inflammation, and extensive degradation of extracellular matrix components. Plaque formation is initiated by the sub-endothelial accumulation of lipids, cholesterol, and other blood components within the intimal layer of the vessel wall [4]. Carotid atherosclerotic plaques comprise both stable and unstable atheromatous lesions [5]. The intrinsic composition of unstable plaques, including a large lipid core, thin fibrous cap, and intraplaque hemorrhage, are associated with the occurrence of cerebral ischemic events [5]. Hypertension, diabetes, and smoking are predictive factors for the development and progression of atherosclerosis and the formation of carotid plaques [6, 7]. These findings highlight the importance of managing these risk factors in the prevention and treatment of cerebrovascular disease.

Carotid atherosclerotic plaque progression is associated with an increased risk of stroke and is responsible for 15–20% of ischemic strokes [8–10]. These plaques are also associated with poor cognitive function [11]. The severity of carotid stenosis is the primary parameter for determining the need for medical or surgical treatments [12, 13]. However, patients with severe carotid stenosis may remain free of cerebral ischemia symptoms in the long term [14, 15], and patients with non-severe carotid stenosis can develop cerebrovascular events and symptoms caused by hypoperfusion due to progressive stenosis and inadequate collateral circulation or thromboembolism caused by the release of thrombogenic necrotic core material due to plaque rupture [16–19].

Ultrasound is currently the first-line imaging method for investigating carotid intima-media thickness, carotid stenosis, and carotid plaque features. The carotid plaque features assessed using B-mode ultrasound, including echogenicity, surface morphology, and plaque size, can as independent parameters for the prediction of cerebrovascular symptoms [20–24]. Fibrous calcified carotid plaques often have an echogenic appearance on B-mode ultrasound images, whereas, plaques that contain lipids and intraplaque hemorrhage are echolucent and at risk of rupturing [20–22]. A study conducted by Elhfnawy et al. (2019) examined the relationship between plaque size and the risk of ischemic stroke and found that large plaques may increase the risk of ischemic stroke [24]. Other studies reported that ultrasonically determined carotid plaque echogenicity and size can independently predict future cerebrovascular events [25, 26]. It has recently been recommended that the morphological features and size of carotid atherosclerotic plaque in addition to the degree of stenosis should be considered for optimal therapeutic or operative decision-making [26]. Therefore, it is important to investigate carotid plaque size causing low-grade stenosis in addition to its morphological characteristics and echogenicity to identify patients who may develop cerebrovascular symptoms or events. We aimed to investigate carotid plaques with low-grade (<50%) stenosis to identify potential biomarkers that can be used to predict future cerebrovascular events or symptoms to aid in treatment decision-making.

## Methods

### Study design and population

This retrospective study was performed on data collected from the archive of King Abdulaziz University Hospital in Jeddah, Saudi Arabia, on patients with carotid disease who underwent carotid ultrasound examination at the Department of Radiology. This study was approved by the Unit of Biomedical Ethics Research Committee of the Faculty of Medicine and the Department of Radiology at King Abdulaziz University and King Abdulaziz University Hospital (reference no: 584–21). In this retrospective study, we reviewed a dataset of 1,593 patients who underwent carotid ultrasound between 2016 and 2021 in January through April of 2022. The data were fully anonymized, and the institutional review board or ethics committee waived the requirement for informed consent. During and after the data collection process, only FT had access to identifiable participant information, which was not utilized during data collection. The data were kept blinded until after the analysis. The inclusion criteria were adult patients with low-grade (<50%) carotid stenosis estimated using ultrasound diameter reduction on two-dimensional B-mode ultrasound and patients with no previous surgical or endovascular interventions. If cerebrovascular events or symptoms were mentioned in the patient’s clinical history records, they were considered symptomatic. Patients with carotid plaque causing ≥50% stenosis, with no carotid plaque following ultrasound examination or those who underwent surgical or endovascular interventions were excluded. All patients who met the inclusion criteria were included in the analysis.

### Data acquisition and ultrasound image analysis

**Fig 1:**
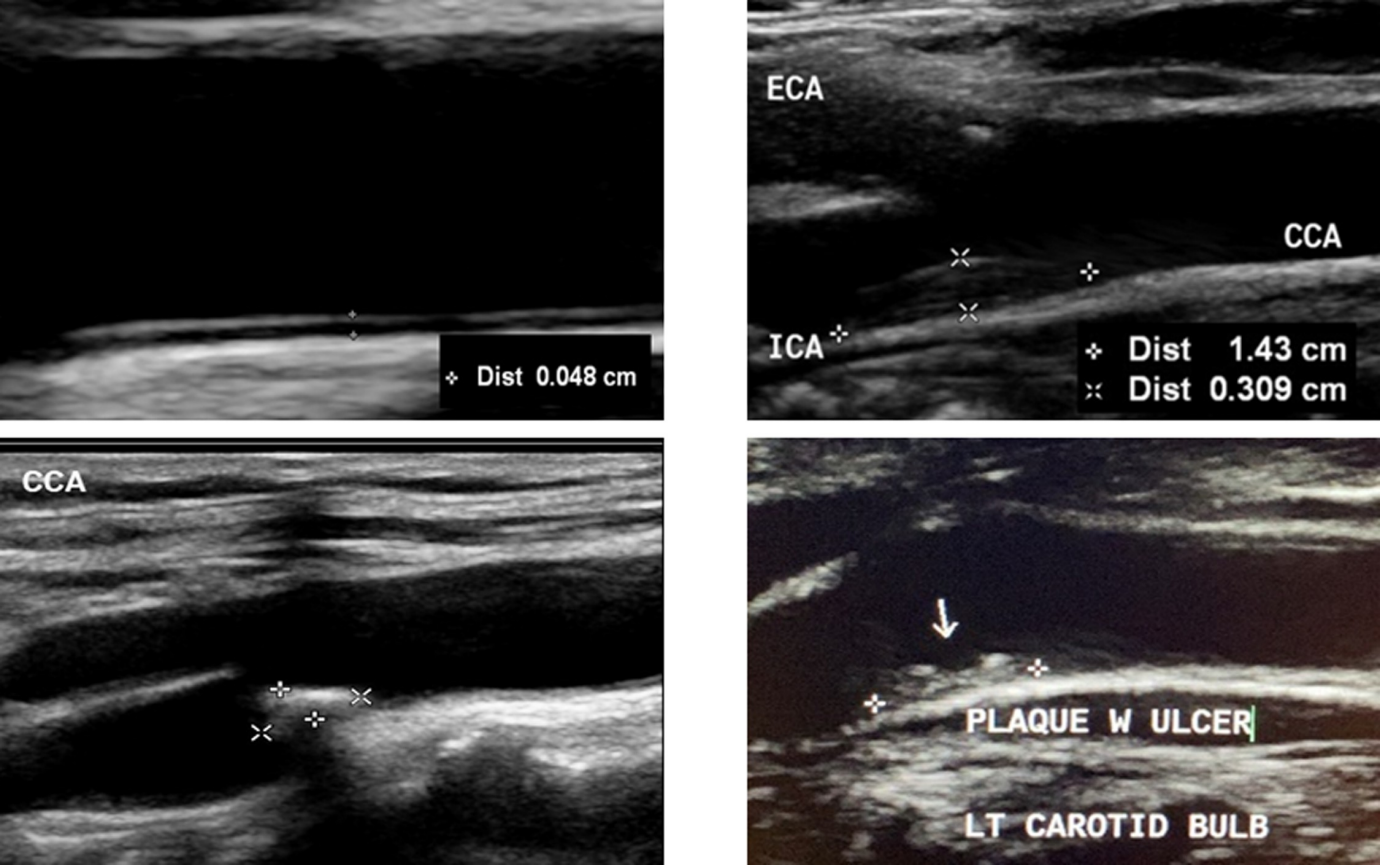
B-mode carotid plaque characteristics. Common carotid artery intima-media thickness (A), a predominately hypoechoic carotid plaque with a smooth regular surface and size (B), predominately hyperechoic plaque (C), and carotid plaque with irregular surface (D).

Patient information and clinical data, including age, gender, chronic diseases (i.e., hypertension and diabetes), smoking, and cerebral symptoms were extracted from the clinical history in the patients’ files. Carotid ultrasound examination was performed on the included patients using a 9–3 MHz broadband linear array transducer of a high-resolution ultrasound imaging system (IU-22, Philips Healthcare, Bothell, Washington, United State of America). The common carotid artery, internal carotid artery, and external carotid artery were assessed in long and short axes bilaterally. Two-dimensional B-mode images and Doppler images including color and spectral Doppler, were acquired. Ultrasound B-mode settings were optimized for high-quality gray-scale images. Depth was adjusted to eliminate tissues underneath the vessel of interest, the focus point was placed at the level of the vessel of interest for better lateral resolution, and the overall gain and time-gain compensation and dynamic range of the image were optimized. Ultrasound images were interpreted and analyzed by two experienced clinical ultrasound specialists who were blinded to the patients’ clinical history. From the B-mode images acquired in longitudinal view, common carotid intima-media thickness (cIMT), plaque size (i.e., length, thickness, and area), degree of stenosis, and morphological features (i.e., echogenicity and surface morphology) were measured and evaluated off-line. cIMT is defined as the distance from the lumen-intima interface to the media-adventitia interface [27]. cIMT is measured in the far wall proximal to the carotid bifurcation at a plaque-free region. The length and thickness of the carotid plaque, a focal structure with a thickness >1.5 mm protruding into the carotid artery lumen [28], were measured, and plaque area (i.e., thickness ×length) was calculated. For echogenicity assessment, plaques were classified through visual assessment as predominantly hypoechoic for a plaque with high lipid and hemorrhage content and predominantly hyperechoic for a plaque rich in fibrous tissue with extensive calcification [22, 29]. Plaque surface morphology was classified as a smooth regular surface or irregular surface, an uneven with high and low fluctuation or with ulceration (Fig 1A–1D) [24, 30, 31] (Fig 1A–1D). If images were not available, the data were extracted from radiology reports.

### Statistical analysis

Statistical analysis was performed using a Chi-square test for the comparison of categorical variables, that is, cIMT, carotid plaque features, patients’ clinical characteristics, and the presence or absence of stroke and/or cerebrovascular symptoms, to identify associations between these variables and the occurrence of cerebrovascular symptoms. Independent t-test and Mann–Whitney U test were used for parametric and non-parametric data, respectively, to compare cIMT and plaque length and thickness to identify significant differences in these variables between the symptomatic and asymptomatic patients. Blinded data analysis was performed using SPSS Statistics (Version 21.0. Armonk, NY: IBM Corp) and PRISM 7 (GraphPad, Software, La Jolla, CA, USA). Statistical significance was set at a p-value less than 0.05.

## Results

### Clinical characteristics and cerebrovascular symptoms

A total of 107 carotid plaques from 87 patients (35 symptomatic and 52 asymptomatic) causing an average of 34.2±12% stenosis (symptomatic, 36.1±11.3%; asymptomatic, 32.5±12.4%, p = 0.1, n = 97) (mean age±standard deviation (SD)) were identified and evaluated using diameter reduction in B-mode ultrasound.. Of the 87 patients included in this study, 65 were male (26 symptomatic and 39 asymptomatic) and 22 were female (9 symptomatic and asymptomatic), 56 had hypertension (28 symptomatic and asymptomatic), 43 were diabetic (19 symptomatic and 24 asymptomatic), 10 were smokers (four symptomatic and six asymptomatic), 44 had suffered from a stroke and cerebrovascular symptoms, and 20 had bilateral plaques (10 symptomatic and 10 asymptomatic). A summary of the patient information is presented in Table 1.

**Table 1:** Patient information.

	Total [*n* = 87]	Symptomatic [*n* = 35]	Asymptomatic [*n* = 52]
**Male, *n* [%]**	65 [74.7]	26 [57.7]	39 [75]
**Age, mean ±SD**	69.58±10.5	68.6.±10.4	69.5±10.6
**Hypertension, *n* [%]**	56 [48.4]	28 [80]	28 [56]
**Diabetes, *n* [%]**	43 [51.2]	19 [54.2]	24 [46.1]
**Smoking, *n* [%]**	10 [88]	4 [11.4]	6 [11.5]
**Stroke, *n* [%]**	9 [10.3]	9 [10.3]	-
**Cerebrovascular symptoms:**	29 [33.3]		-
**Confusion, *n* [%]**		1 [2.8]	
**Limb weakness, *n* [%]**		28 [80]	
**Trouble speaking, *n* [%]**		3 [8.5]	
**Dizziness, *n* [%]**		5 [14.2]	
**Numbness, *n* [%]**		4 [11.4]	
**Patient with bilateral plaques, *n* [%]**	20 [22.9]	10 [28.5]	10 [19.2]

Abbreviation: *n*: number of patients, SD: standard deviation

**Fig 2:**
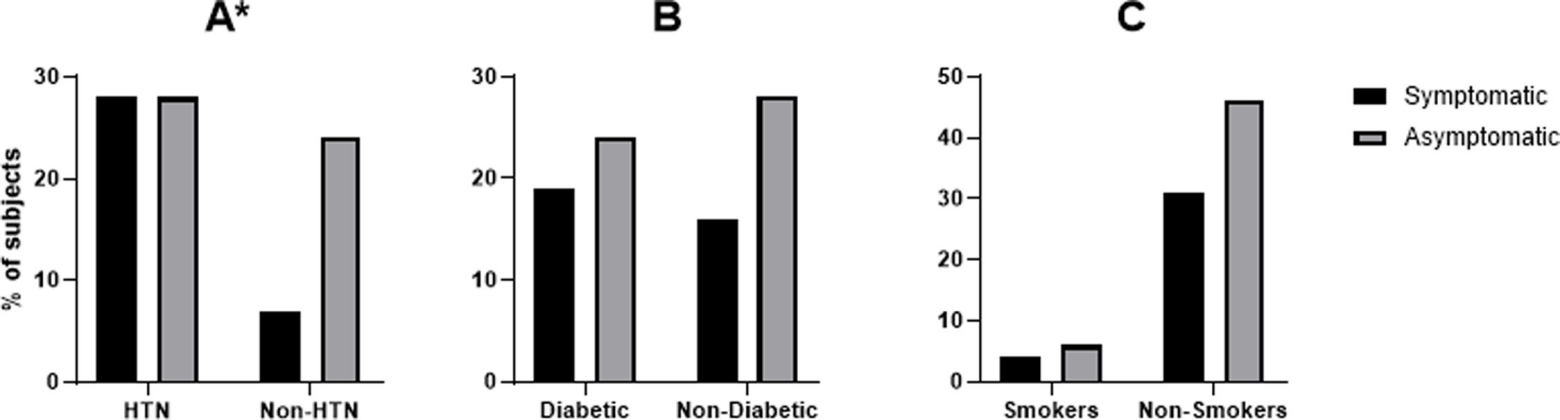
Clinical characteristics in symptomatic and asymptomatic patients with low-grade carotid stenosis. Hypertensive vs non-hypertensive patients (HTN, A), diabetic vs non-diabetic patients (DM, B), and smokers vs non-smokers patients (C). *p<0.05 using chi-square test.

There was no significant difference in age between symptomatic and asymptomatic patients (symptomatic: 68.6±10.4 years; asymptomatic: 69.5±10.6 years, p = 0.7) (mean age±SD). There was a significant association between hypertension and the presence of cerebrovascular symptoms (Pearson`s Chi-square 6.23, p = 0.01, Fig 2A). Symptomatic patients with carotid plaque were more likely to be diagnosed with hypertension than in asymptomatic patients, with 80% to 53.8%. There was no significant associations between diabetes, smoking cigarette, and the presence or absence of cerebrovascular symptoms (diabetes: p = 0.45, Fig 2B; smoking cigarettes: p = 0.9, Fig 2C).

### Carotid plaque features and cerebrovascular symptoms

**Fig 3:**
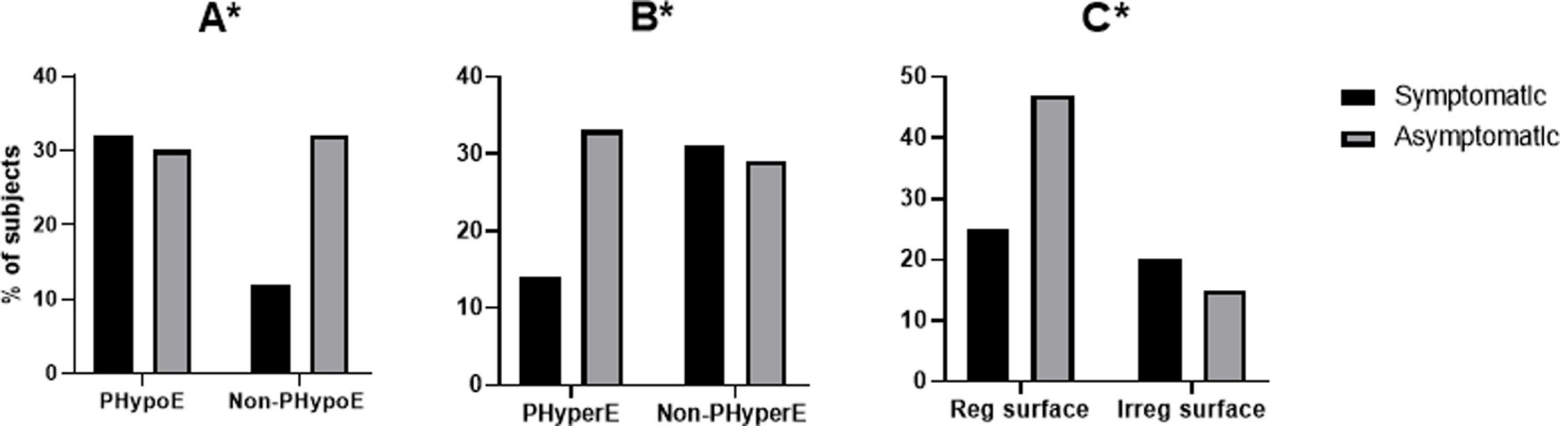
Carotid plaque echogenicity and surface regularity in symptomatic and asymptomatic patients with low-grade stenosis. Predominantly hypoechoic plaque (PHypoE, A), predominantly hyperechoic plaque (PHyperE, B), and surface regularity (C). * p<0.05 using the Chi-square test. PHypoE: predominantly hypoechoic plaque, PHyperE: predominantly hyperechoic plaque, Reg: regular, Irre: irregular.

Predominantly hypoechoic and hyperechoic carotid plaques were significantly associated with the presence and absence of cerebrovascular symptoms, respectively (predominantly hypoechoic: Pearson’s Chi-square 6.23, p = 0.01, n = 107, Fig 3A; predominantly hyperechoic: Pearson’s Chi-square 6.23, p = 0.01, n = 107, Fig 3B). Predominantly hypoechoic carotid plaques were more likely to be observed in symptomatic patients than in asymptomatic patients, with 80% to 53.8%. In contrast, predominantly hyperechoic plaques were more likely to be detected in asymptomatic patients than in symptomatic patients, with 80% to 53.8%. Surface irregularity was significantly associated with the presence of cerebrovascular symptoms (Pearson’s Chi-square 4.85, p = 0.02, n = 107, Fig 3C) and was more frequently detected in the carotid plaques of symptomatic patients than in asymptomatic patients, with 44.4% to 24.2%.

**Fig 4:**
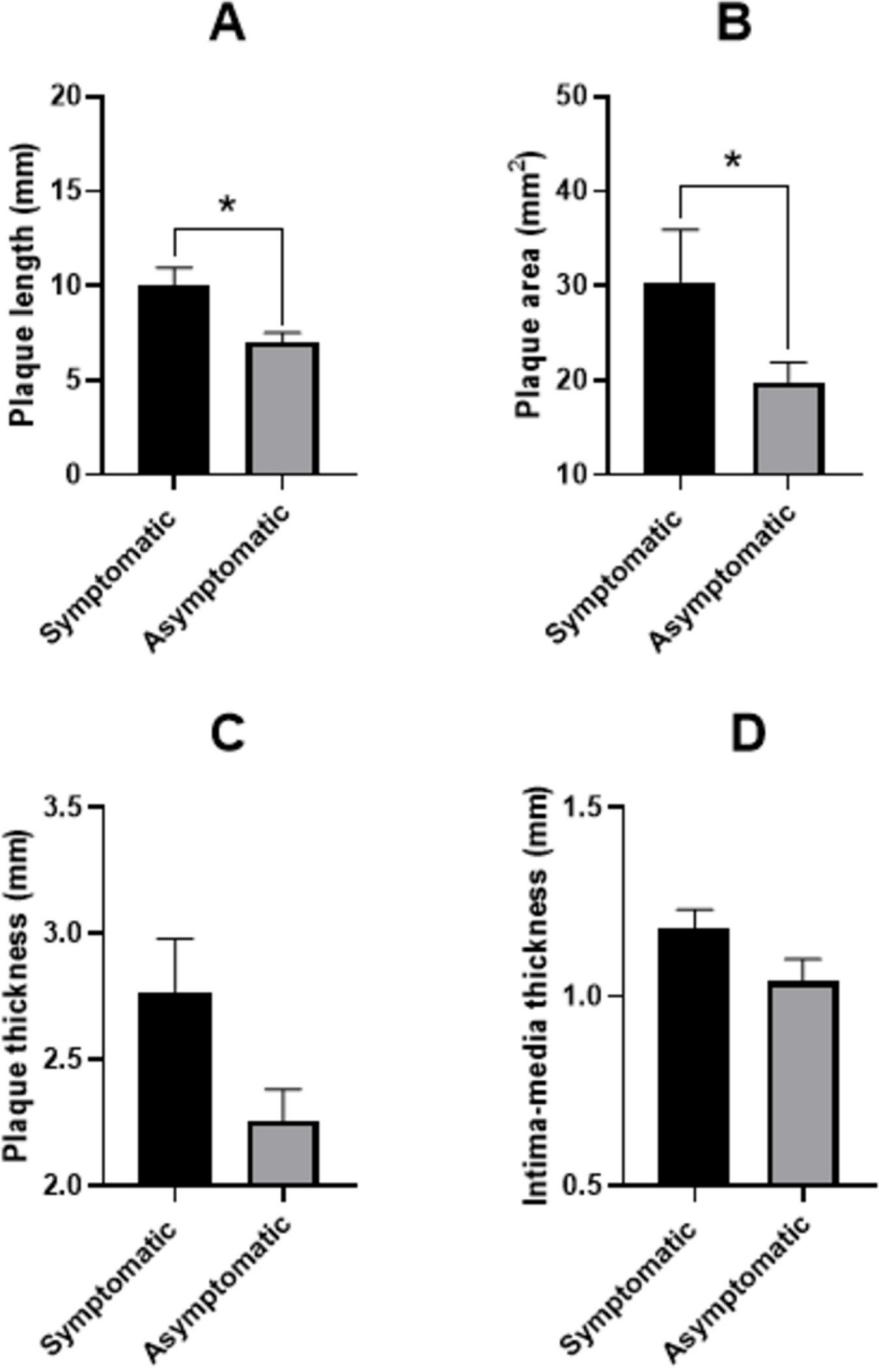
Carotid plaque length (A), area (B), thickness (C), and intima-media thickness (D) in symptomatic and asymptomatic patients with low-grade stenosis. * p<0.05 using Mann-Whitney U test (mean±SEM).

There was a significant difference in carotid plaque length and area between symptomatic and asymptomatic patients (plaque length: symptomatic median: 9 mm, interquartile range [IQR] 6 mm; asymptomatic median: 6 mm, IQR 4.5 mm, z = -2.5, p = 0.01, n = 101, Fig 4A; plaque area: symptomatic median: 24 mm, IQR 30 mm; asymptomatic median: 14 mm, IQR 17 mm, z = -2.3, p = 0.01, n = 102, Fig 4B); however, difference in plaque thickness (p = 0.55, n = 104, Fig 4C), and cIMT (p = 0.1, n = 99, Fig 4D) were not statistically significant. Plaque features in symptomatic and asymptomatic patients are presented in Table 2.

**Table 2:** Features of carotid plaques.

	Symptomatic [*n* = 45]	Asymptomatic [*n* = 62]	*Probability of a difference*
**DoS % [mean±SD]**	36.1±11.3	32.5±12.4	*t*_(97)_ = -1.4; *p =* 0.1
**cIMT in mm [mean±SD]**	1.2±0.2	1.1±0.3	*t*_(99)_ = -0.2_*;*_ *p* = 0.7
**Plaque length in mm [median, IQR]**	9, 6	6, 4.5	Z_(101)_ = -2.5; *p =* 0.01
**Plaque thickness in mm [median, IQR]**	3, 1.6	3, 1	Z_(104)_ = -0.5; *p =* 0.55
**Plaque area [median, IQR]**	24, 30	14, 17	Z_(102)_ = -2.3; *p =* 0.01
**Surface irregularity, *n* [%]**	20 [44.4]	15 [25]	X^2^_(107)_ = 4.8; *p =* 0.02
**Predominately hypoechoic, *n* [%]**	31 [68.8]	29 [46.7]	X^2^_(107)_ = 6.2; *p =* 0.01
**Predominately hyperechoic, *n* [%]**	14 [31.1]	33 [53.2]	X^2^_(107)_ = 6.2; *p =* 0.01

Abbreviation: cIMT: common carotid intima-media thickness, DoS: degree of stenosis, IQR: interquartile range, *n*: number of carotid plaques, SD: standard deviation.

## Discussion

The indication for surgical or endovascular treatment is largely determined by the severity of carotid stenosis in symptomatic and asymptomatic patients. However, patients with non-severe carotid stenosis may develop cerebral symptoms that require operative intervention [32]. In the present study, we investigated the morphological features and clinical characteristics of carotid plaques from patients with low-grade (<50%) as an attempt to predict potential future cerebrovascular symptoms and help proactive treatment decision-making. Hypertensive patients with predominantly hypoechoic plaques with an irregular plaque surface and those with large carotid plaques were more likely to suffer from cerebral symptoms or events. These findings suggest that a patient’s characteristics and carotid plaque features, including echogenicity, surface irregularity, and plaque size, should be considered in addition to the severity of carotid stenosis for optimal treatment selection.

Hypertension and diabetes are the most important risk factors for cerebrovascular complications, and they can cause stroke by altering cerebral vessel structure [33–35]. Hypertension is a primary risk factor for all types of stroke [3], and an isolated increase in systolic blood pressure is frequently observed in patients with cerebrovascular complications [36]. An epidemiological study found that decreasing high blood pressure is a major influencing factor on the decline in stroke death rate [37]. Hypertension leads to endothelial dysfunction, oxidative stress, and inflammation, which can promotes the formation and growth of carotid plaques [38]. Similarly, diabetes can exacerbate the progression of carotid plaque by promoting dyslipidemia, hyperglycemia with advanced glycation end-product formation, increased oxidative stress, and inflammation [39]. Cigarette smoke contains numerous toxic chemicals that can promote the build-up of plaque, including nicotine and carbon monoxide, which contribute to oxidative stress, the upregulation of inflammatory cytokines, endothelial dysfunction, and plaque formation [40]. However, a study assessing the histological features of symptomatic carotid plaques in relation to smoking reported that plaque morphology was similar in smokers and ex- or never-smokers, suggesting that plaque vulnerability is unrelated to smoking [41]. In the present study, we found no association between patients who were symptomatic and diabetics or cigarette smokers, which may be explained by the inclusion of patients with controlled diabetes or those who were recently diagnosed with diabetes [42]; furthermore, the proportion of cigarette smokers in our cohort was only 11.5%. Larger studies investigating clinical risk factors associated with cerebrovascular symptoms and stroke in patients with carotid diseases are warranted.

Several studies assessing the use of B-mode ultrasound to characterize carotid plaque echogenicity reported that predominately hypoechoic plaques often contain lipids and intraplaque hemorrhage and are at risk of rupturing; in contrast, predominately hyperechoic plaques are considered stable as they contain fibrous and calcified tissue [20–22, 43]. In our present study, we observed predominantly hypoechoic carotid plaques significantly more frequently in patients with cerebral symptoms and adverse events, while hyperechoic plaques were more common in asymptomatic patients. In addition, we also found that plaque surface irregularity, length, and area, but not plaque thickness were associated with the presence of cerebral symptoms. Several studies have demonstrated a significant correlation between the presence of atherosclerotic plaques with an irregular surface and cerebral symptoms [23, 44, 45], with a reported association between carotid plaque length and area and the presence of micro-emboli and increased risk of ischemic stroke or transient ischemic attack in patients with low-grade carotid stenosis [25, 46]. It has been reported that the growth of a carotid plaque’s length is more rapid than its corresponding thicknesses [46, 47], suggesting that plaque area is more influenced by the length of the plaque. Furthermore, our analysis revealed no significant difference in cIMT between symptomatic and asymptomatic patients. It has been reported that cIMT is not associated with markers of atherosclerosis in stroke patients and must only be considered a risk factor for general vascular disease [48]. This suggests that plaque surface irregularity, length, and area may be indicators for stroke risk stratification in non-severe carotid stenosis.

Computer-assisted analysis and advanced vascular imaging techniques can be used to identify additional parameters to better evaluate and quantify the morphological features of stable and unstable carotid plaques [43, 49]. The use of computer-assisted analysis to determine carotid plaque echogenicity through the gray-scale median score by analyzing the echogenicity of a carotid plaque on a B-mode ultrasound image suggests that low median gray-scale values (hypoechoic) are associated with higher lipid content and inflammatory cells, while plaques with high gray-scale median values (hyperechoic) are associated with increased calcium composition [50–52]. A recent systematic review and meta-analysis conducted by our group on the use of contrast-enhanced ultrasound for the evaluation of carotid plaque revealed significantly higher contrast-enhanced ultrasound parameters in patients with symptomatic carotid plaques compared with asymptomatic plaques, suggesting that contrast-enhanced ultrasound is superior to B-mode for quantifying carotid plaque neovascularization [53]. In addition, it has been reported that 3D ultrasound is a useful method for quantifying carotid plaques by measuring carotid plaque volume, which correlates with symptoms of cerebral ischemia [54–56]. These observations suggest that ultrasound is a useful imaging method that can differentiate symptomatic from asymptomatic carotid plaque. Further studies investigating symptomatic and asymptomatic carotid plaque features using multiparametric ultrasound and their correlation with histology as a gold standard are required.

Our present study had several limitations to consider when interpreting the results. The sample size was relatively small which could have been related to not observing the association between traditional risk factors (i.e., diabetes and smoking) and symptomatic patients. Further studies with larger sample sizes investigating risk factors associated with the development of carotid plaques and cerebrovascular symptoms are required. It is important to consider that this study was conducted retrospectively and that the assessment of symptoms relied solely on chart review, which may have implications for the interpretation of the results. In addition, it would be valuable to investigate the duration of follow-up after carotid ultrasound in future studies. The duration of chronic diseases, medications, and lifestyle, including food, diet, and exercise, may impact the risk of developing cerebrovascular symptoms in patients with carotid disease [57, 58]. In addition, there are marked differences in atherosclerotic cardiovascular disease risk in different ethnic groups [59], which can affect the generalizability of the study results. However, these factors are beyond the scope of the present study and were not assessed. An intensive investigation of these factors could provide a more comprehensive understanding of the relationship between carotid plaque morphological features, clinical characteristics, and the development of cerebrovascular symptoms. Doppler parameters of carotid arteries were not investigated; we included only patients with low-grade stenosis in which significant changes in hemodynamic and Doppler parameters may not be evident. Although this study provided insight into the potential use of the morphological features of carotid plaques and clinical characteristics to predict the development of cerebrovascular symptoms in patients with low-grade carotid stenosis, future studies should address these limitations and provide more comprehensive investigations into the relationship between the morphological features of carotid plaques, clinical characteristics, and the development of cerebrovascular symptoms.

## Conclusion

Investigating effective and reliable biomarkers for predicting future cerebrovascular symptoms and adverse events is essential. Hypertensive patients with a predominantly hypoechoic plaque with an irregular surface, or a large carotid plaque might represent a marker of increased risk for further complications of cerebrovascular diseases. These features can be useful for proactive treatment of patients with low-grade carotid stenosis. Large prospective studies stratifying the risk of cerebrovascular symptoms and events associated with high-risk clinical characteristics including chronic diseases, medications, food, diet, exercise, ethnicity, and carotid plaque features in patients with different grades of stenosis are required.

## Supporting information

S1 File Patient information. (XLSX)

S2 File Features of carotid plaques. (XLSX)
